# Supplementary material for: High Frequency of Non-Compliance with Quality Indicators of Enteral and Parenteral Nutritional Therapy in Hospitalized Patients
Source: Nutrients. 2020 Aug 12;12(8):2408. doi: 10.3390/nu12082408 (PMC7468971; doi:10.3390/nu12082408)
Supplement: Supplementary file 1 [file nutrients-12-02408-s001.pdf]

Table S1. Quality indicators in enteral and parenteral nutritional therapy, formulas and proposed goals.

Indicator	Formula	Goal (%)
Frequency of carrying out nutrition screening	<u>Nº of screening nutrition in 24 hours x 100</u><br>Total no. of hospitalizations	>80
Frequency of application of SGA in patients on EN and PN	<u>Nº of patients with SGA in EN and PN x 100</u><br>Total no. of patients in EN and PN	>75
Frequency of nutritional reassessment in patients on EN	<u>Nº of patients reassessed in EN in the last 7 days x 100</u><br>Total no. of patients in EN	>80
Frequency of compliance of indication of EN	<u>Nº of patients on EN indicated according guidelines x 100</u><br>Total no. of patients on EN	<13.5
Frequency of measurement or estimation of energy expenditure and protein requirement in patients on EN and PN	<u>Nº of patients on EN and PN with measurement energy expenditure and protein x 100</u><br>Total no. of patients on EN and PN	>80
Frequency of adequacy of prescribed EN volume versus administered	<u>Nº of patients on EN with infusion as prescribed x 100</u><br>Total no. of patients in EN	>90
Frequency of digestive fasting for more than 24 hours in patients on EN	<u>Nº of patients in fasting > 24 hours x 100</u><br>Total no. of patients on EN	<12
Frequency of involuntary withdrawal of enteral feeding tube	<u>Nº of involuntary withdrawal of feeding tubes x 100</u><br>Total no. of patients on EN x N° of days with feeding tube	<10
Frequency of tube feeding occlusion in patients on EN	A) <u>Nº of tube feeding occlusion in patients on EN x 100</u><br>Total no. of patients on EN x N° of days with feeding tube<br>B) <u>Nº of tube feeding withdrawal per occlusion on EN x 100</u><br>Total no. of patients on EN x N° of days with feeding tube	<5
Frequency of CVC infection in patients on PN	<u>Nº of occurrences of infection associated with CVC x 100</u><br>Nº of days of catheter	CVC- via peri (PIC): infection with bacteremia < 2,5<br>CVC: infection without bacteremia < 10<br>CVC: infection with bacteremia < 5
Frequency of patients with glycemic dysfunction on EN and PN	<u>Nº of patients with hypoglycemia x 100</u><br>Total no. of patients on EN, PN or both<br><u>Nº of patients with hyperglycemia 100</u><br>Total no. of patients on EN, PN or both	**Hyperglycemia:**<br>Noncritically ill: 10%–30%<br>**Hypoglycemia:** 5,1%–6,9%
Frequency of diarrhea in patients on EN	A) <u>Nº of patients on EN with diarrhea x 100</u><br>Total no. of patients on EN<br>B) <u>Nº of days with diarrhea and EN x 100</u> (Per patient)<br>Total no. of days on EN	<10

Abbreviation: EN, enteral nutrition; PN, parenteral nutrition; SGA, subjective global assessment; CVC, central venous catheter; PIC, peripheral intravenous catheter.
Adapted from Verotti et al, 2012.
